# Caring for Those Who Take Care of Others: Developing Systemic and Sustainable Mental Health Support for the Diverse Healthcare Workforce in the United Kingdom

**DOI:** 10.3390/ijerph20043242

**Published:** 2023-02-13

**Authors:** Irtiza Qureshi, Jonathan Chaloner, Mayuri Gogoi, Amani Al-Oraibi, Fatimah Wobi, Holly Reilly, Asta Medisauskaite, Christopher A. Martin, Patricia Irizar, Padmasayee Papineni, Susie Lagrata, Joy Agbonmwandolor, Manish Pareek, Laura Nellums

**Affiliations:** 1 Lifespan and Population Sciences, School of Medicine, University of Nottingham, Nottingham NG7 2RD, UK; 2 Department of Respiratory Sciences, University of Leicester, Leicester LE1 7RH, UK; 3 Public Health Institute, Faculty of Health, Liverpool John Moores University, Liverpool L3 5UX, UK; 4 Research Department of Medical Education, UCL Medical School, London WC1E 6DE, UK; 5 Department of Sociology, School of Social Sciences, University of Manchester, Manchester M13 9PL, UK; 6 Department of Infectious Diseases, London North West University Healthcare NHS Trust, London UB1 3HW, UK; 7 The National Hospital for Neurology and Neurosurgery, London WC1N 3BG, UK; 8 The David Evans Medical Research Centre, Nottingham University Hospital NHS Trust, Nottingham NG5 1PB, UK

**Keywords:** workforce, mental health, intervention, occupational health, organizational policy

## Abstract

Pressures such as high workload, stretched resources, and financial stress are resulting in healthcare workers experiencing high rates of mental health conditions, high suicide rates, high rates of staff absences from work, and high vacancy rates for certain healthcare professions. All of these factors point to the fact that a systematic and sustainable approach to mental health support at different levels and in different ways is more important than ever. In response, we present a holistic analysis of the mental health and wellbeing needs of healthcare workers across the United Kingdom healthcare ecosystem. We recommend that healthcare organisations should consider the specific circumstances of these staff and develop strategies to counter the negative impact of these factors and help safeguard the mental health of their staff.

## 1. Introduction

**Figure 1:**
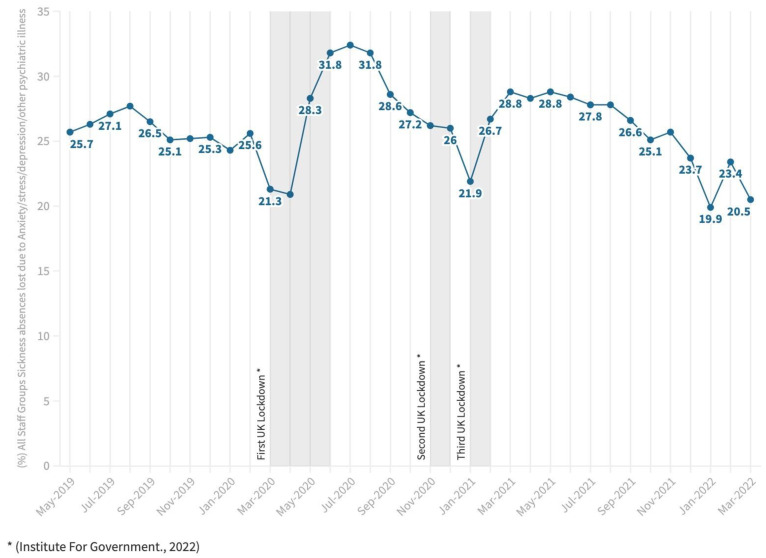
All staff groups sickness absence as a percentage of Full-Time-Equivalent (FTE) days lost due to anxiety/stress/depression/other psychiatric illnesses [1,6].

Even before the COVID-19 pandemic, it was recognised that in the United Kingdom (UK), National Health Service (NHS) healthcare workers (HCWs) were consistently experiencing high rates of mental health conditions including anxiety, stress, and depression, resulting in high rates of staff absences from work (see Figure 1) [1] Other pressures for HCWs include high workload and extremely stretched resources [2]. Specific research in the UK has shown substantial levels of probable common mental disorders and post-traumatic stress disorder with lower levels of depression, anxiety, and alcohol misuse within HCWs in the first wave of the pandemic [3].

Higher suicide rates are also a consequence of the long-established higher psychological burden and mental stress placed on HCWs than other professions [4] which have only been exacerbated by the pandemic [5]. All of these factors point to the fact that a systematic and sustainable approach to mental health support at different levels and in different ways is more important than ever.

Even though the prevalence rates for various mental health conditions and absence rates from places of work may have fluctuated and be subject to differing analyses over the different waves of the pandemic [7], it is critical that all HCWs are appropriately supported and protected from mental harm, as the World Health Organization (WHO) has justifiably named them as ‘our most valuable resource for health’ [8]. Specific research into current and recommended methods for HCWs’ mental health management in relation to the pandemic has acknowledged the importance of this issue. Such research highlights the need for mangers to acts as role models leading by example and taking time out for their own wellbeing; giving psychological permission to HCWs ‘not to be ok’; personalise communications with teams; refresh their active listening skills and prioritise reflexive time and space for HCWs to process demanding work events at the individual and team level [9]. However, more needs to be done at multiple levels to better manage the mental health needs of HCWs in the UK.

**Figure 2:**
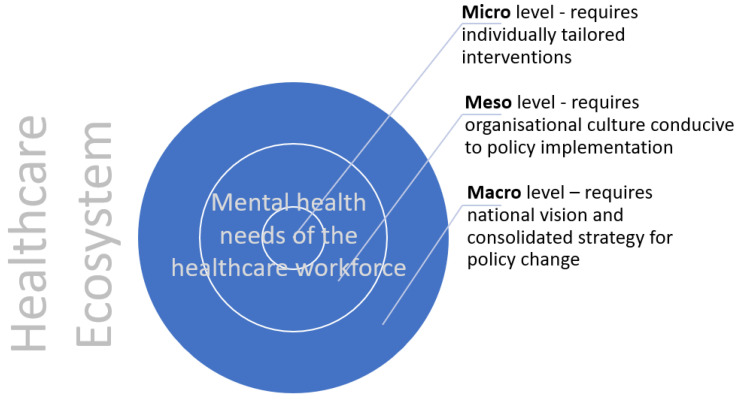
Healthcare ecosystemic model of mental health needs of healthcare workers.

The healthcare ecosystem, at macro-, meso-, and micro-levels, needs to better protect their mental health, which requires a sustainable and systemic approach integrating mental health support (see Figure 2). This approach is particularly relevant for marginalised staff, in the context of the extensive research reporting the institutional and interpersonal racism, discrimination, and disadvantage that ethnic minority staff face within the NHS [10,11,12]. Equity, providing resources based on the needs of HCWs rather than equality, which gives everyone the exact same resources, is required in this approach [13].

## 2. Discussion

At a macro-level, the UK Government has set out some strategic aspects of how it plans to support NHS staff mental health and wellbeing. This includes the launch of ‘Build Back Better: Our Plan for Health and Social Care’, in March 2022 [14]. This policy paper came as an amalgamation of initiatives from three different government departments: Cabinet Office, Department of Health and Social Care, and the Prime Minister’s Office. It states that in order to tackle the backlog of patients, with waiting times of over 40% longer than before the pandemic, the government plans to ‘increase and make best use of available resources’ [14], meaning that, until the longstanding NHS vacancies are filled (over 50,000 nurses and over 8000 doctors are required), the government will rely upon existing staff members to address the backlog. This is likely to result in extra hours worked by staff members working prior to the pandemic in 2019, for whom it was reported that 53.9% of NHS staff were already experiencing high rates of excessive and conflicting demands on workloads [15]. However, we also know that having to work longer hours than usual is already a major determinant of work-related stress and burnout [16] and is subsequently associated with increased occupational and patient safety risks as well as mistakes [17]. The cumulative pressures from poor management factors such as negative leadership behaviours, work-load expectations, insufficient rewards, limited interpersonal collaboration, and limited opportunities for advancement and social support from managers add to their pressures. This is in addition to work pressures such as long working hours, specialty choice, frequent call duties, comprehensive documentation in electronic medical records, time spent at home on work-related factors, risk of malpractice suits and patient death, and illness. There are personal traits such as being overly self-critical, engaging in unhealthy coping strategies, sleep deprivation, overcommitment, perfectionism, idealism, work–life imbalance, and an inadequate support system outside the work environment which also contribute to their mental burden [18].

Without the additional staff members and necessary investment in new technologies for digital health, these desired outcomes simply will not happen, resulting in increased risk of burnout, which will further shrink the workforce. Again, the link between diminished and psychologically burdened healthcare workforces and poor quality and safety in healthcare delivery has already been established in prior research [19,20].

In the national guidance launched in May 2022 ‘COVID-19 Response: Living with COVID-19’, which aims to support the NHS going forward, the government is committed to ‘implementing a range of workforce interventions’ including increasing staffing numbers, temporary local adjustments to staffing ratios, with flexible redeployment of staff including training for roles in critical or enhanced care’ [21]. We have shown that employing organisations have lacked operational capacity to deliver usual levels of care, which has meant existing staff having to take up extra work and responsibilities coupled with extra patients, adding to their mental distress [22]. This concept of flexible redeployment is particularly concerning for ethnic minority staff who reported disproportionate redeployment to COVID-19 ‘hot wards’ [23] at times without adequate training [24].

The most telling of all the macro-policy responses has been ‘The government response to the Health and Social Care Committee (HSCC) report on workforce burnout and resilience in the NHS and social care’, published in February 2022 [25]. This policy paper aims to directly address the issues at the heart of preventing healthcare staff burnout, but also improve ‘resilience’ within the health system to withstand, respond to, and recover from challenging scenarios [26].

Key recommendations from the initial HSCC report included that the level of resources allocated to mental health support for health and care staff be maintained as and when the NHS and social care return to ‘business as usual’ after the pandemic, and that the adequacy of resources allocated to that support be monitored on a regular basis.

The disappointing government response stated that as part of the government’s White Paper, ‘People at the Heart of Care: Adult Social Care Reform’, published 1st December 2021, at least GBP 500 m of investment was announced for the workforce. A part of that will fund mental health and wellbeing resources, as well as access to occupational health funding; however, only £1 million had been invested in specific wellbeing interventions for the health and adult social care workforce, to build on mechanisms that some employers already have in place. This included the production of wellbeing resources for the NHS workforce including support helplines, wellbeing guidance and bereavement resources, and a package of support for registered managers including a series of webinars and a dedicated advice line. Considering that there are over 1.3 million staff in the NHS, £1 million in investment seems woefully inadequate. Members of The Royal College of Nursing recently going on strike for better pay (the first time in their history) indicates the seriousness of the current situation where HCWs do not feel they are appropriately paid. The issue of financial stress for HCWs manifests itself in different ways from the real-terms drop in wages for nurses, to increased pension taxes for consultants through to cuts to bank staff wages in some organisations.

Importantly, the HSCC report highlights the disproportionate impact the pandemic has had on staff from ethnic minority backgrounds, and emphasises that the treatment of ethnic minority staff too often falls short of ‘the high standards that all staff should rightfully expect’. The HSCC signposted the Public Health England and Black, Asian, and Minority Ethnic (BAME) Communities Advisory Group reports on the subject, which set out a series of actions to address this problem [27]. In addition, the HSCC demanded that the government set out how it plans to implement those recommendations, with a corresponding timeframe. The government response to this request, however, seems to be a conflation of initiatives to tackle vaccine hesitancy and misinformation within specific communities in the population. Rather than tackle the issues head-on for ethnic minority NHS staff, the government delayed immediate action by consulting The Equality and Human Rights Commission (EHRC), who launched an inquiry into racial inequality in health and social care workplaces. The EHRC found that ethnic minority HCWs were at a disadvantage in a number of ways including commissioning and outsourcing policies leading to poor pay and insecure work and fear of raising concerns and a lack of mechanisms to do so. The EHRC calls for urgent action to recognise and address the issues raised in their report; however, we know from previous research that these recommendations are often not acted upon. The Race Report [28] found 589 different recommendations made by previous race and inequality reports since the 1980s, many of which have not been considered.

However, in terms of general interventions for HCWs’ mental health and wellbeing, there are more encouraging developments at the meso-level such as the development of 40 mental health hubs, operating across Integrated Care Systems (ICS).

**Figure 3:**
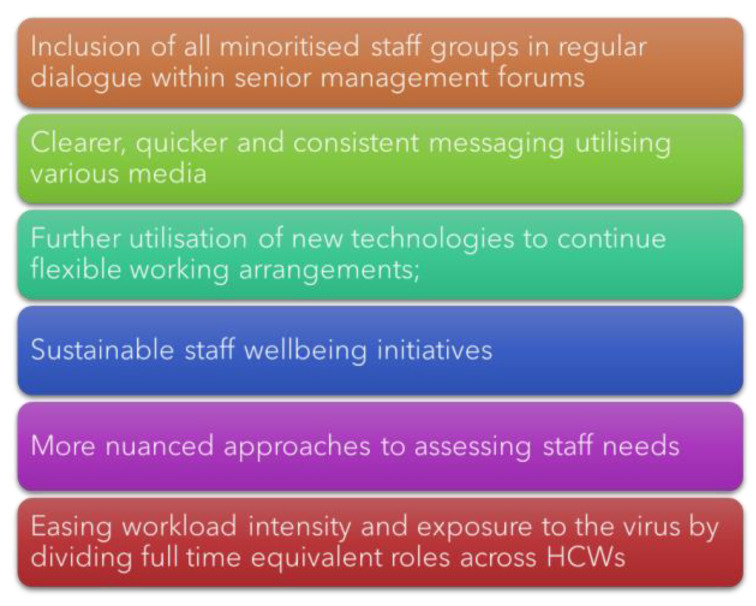
HCW recommendations adapted from [22].

At a micro-level, HCWs are facing issues with not having their basic needs met, including a lack of clean facilities for changing into workwear, lack of affordable free car parking at work, poor-quality IT systems, not enough computers, and inadequate staffing at all levels. In our research from the United Kingdom Research study into Ethnicity And COVID-19 (UK-REACH), HCWs from diverse ethnic backgrounds have made clear policy recommendations to sustainably and systematically address the mental health needs of HCWs (see Figure 3 below).

## 3. Conclusions

The above figure summarises the HCW recommendations for the way forward from our research. Further detailed strategies based on these may include:

1. **Inclusion** of all minoritised and underserved staff groups in key decision-making policies and dialogues at all levels within the policy eco-system.
2. **Increase government-led research and funding** targeted at improving the communication channels and dissemination of information related to policy and guideline changes to HCWs during pandemics and other dynamic national emergencies to reduce the burden of stress and anxiety associated with poor and confusing communications.
3. **Continue to invest** in new technologies to help enable HCWs more flexible working arrangements to facilitate and improve the mental wellbeing of staff with families and encourage the regular taking of paid breaks.
4. **Ensure that all NHS staff** have equal and equitable access to free, flexible, and high-quality mental health services regardless of their speciality, geographical area, and/or shift time. Simultaneously also equip leaders and line-managers to support staff with wellbeing and mental health needs through trainings and resource dissemination.
5. **Nuanced approaches to assessing staff needs** taking into consideration their intersecting social identities and positions (e.g., a female nurse who is also a single mother) are needed to tailor effective mental health interventions and initiatives.
6. **Maximise investments into a sustainable recruitment campaign** to improve and ease workload intensities across all departments to improve the health system’s resilience and response during a future pandemic, combined with reduced post-pandemic financial costs associated with backlogged and delayed elective surgeries.

Our UK-based research aligns with aspects of the international evidence base considering HCW wellbeing and performance which recommend strategies such as systematic application of evidence-based interventions, including mindfulness training, assertiveness training, facilitated discussion groups, and promoting a healthy work environment [18]. This is alongside recommendations for relevant human resources departments and managers to recognise the relationships between work characteristics, mental health issues (sleeping trouble and psychological wellbeing), and physical health issues (fatigue and muscle aches) that influence presenteeism [29].

It is apparent that the current UK governmental response to the mental health needs of NHS and social care key workers is lacking across the ecosystem. At a macro-level, the HSCC’s work highlights NHS staff needs, to which the response has been disparate and inadequate. At a meso-level, time will tell whether the embryonic ICSs will facilitate sustainable wellbeing support for NHS staff. At a micro-level, many of the needs and recommendations set out in the literature (including our research) have yet to be considered in a consolidated way. The Mental Health Foundation’s recent national-level research [30] ‘Coronavirus: Mental Health in the Pandemic Study’ found that the public urgently wanted to see a clear post-Covid vision from the government as a priority. It seems this lack of vision is dangerously missing for those who need it most: those who take care of others.

## Data Availability

Not applicable.
